# Model species driving conservation technologies: twenty years of reproductive research for neotropical wildlife conservation

**DOI:** 10.1590/1984-3143-AR2026-0131

**Published:** 2026-09-21

**Authors:** Alexandre Rodrigues Silva, Luana Grasiele Pereira Bezerra, Romário Parente dos Santos, Andréia Maria da Silva, Gabriela Liberalino Lima Melo, Ana Liza Paz Souza

**Affiliations:** 1 Universidade Federal Rural do Semi-Árido – UFERSA, Mossoró, RN, Brasil; 2 Instituto Federal do Ceará – IFCE, Crato, CE, Brasil

**Keywords:** biobanking, assisted reproduction techniques, caatinga, germplasm preservation

## Abstract

Over the last two decades, reproductive research conducted primarily in Neotropical wildlife species from the Brazilian Caatinga has evolved from descriptive studies of reproductive biology into an integrated framework for biodiversity conservation. The trajectory described herein illustrates how basic reproductive physiology, when systematically linked to assisted reproductive technologies (ARTs), can generate practical conservation tools. Initial investigations focused on reproductive anatomy, endocrinology, spermatogenesis, follicular dynamics, and reproductive behavior. These studies provided the foundation for the development of species-specific methods for semen collection, sperm evaluation, estrous monitoring, hormonal manipulation, cryopreservation, *in vitro* culture, and xenotransplantation. Subsequent advances expanded conservation efforts from gamete preservation to gonadal tissue biobanking, ultimately supporting the establishment of integrated genetic resource conservation strategies. Using species from the Caatinga as experimental models, including collared peccaries, red-rumped agoutis, Spix’s yellow-toothed cavies, six-banded armadillos, greater rheas, and other taxa, this research program generated knowledge applicable to a broad range of Neotropical wild species. Beyond summarizing scientific achievements, this article presents lessons learned during twenty years of research and discusses future opportunities involving advanced biobanking and the use of emerging assisted reproduction technologies. The experience demonstrates that long-term, species-centered programs can serve as effective pathways for transforming biological knowledge into conservation solutions.

## Introduction

Brazil harbors one of the richest biodiversities on Earth, encompassing approximately 701 mammalian species, 1,919 birds, 773 reptiles, and more than 4,500 fish species (Marques, 2023). Preserving this biological heritage is essential not only for maintaining ecosystem functioning and trophic interactions but also for safeguarding the evolutionary potential of natural populations. However, habitat loss, fragmentation, climate change, emerging diseases, and other anthropogenic pressures have accelerated population declines in numerous species, creating an urgent need for effective conservation strategies that integrate both in situ and ex situ approaches (Pizzutto et al., 2021).

Among the various tools available for biodiversity conservation, assisted reproductive technologies (ARTs) and genetic resource banking have emerged as powerful approaches for preserving genetic diversity and supporting population management (Silva et al., 2026). Nevertheless, the successful application of these technologies in wildlife remains limited by a fundamental challenge: the lack of species-specific reproductive knowledge. For many wild species, even basic information regarding reproductive physiology, gamete biology, endocrine regulation, reproductive behavior, or gonadal function remains scarce or completely unavailable. As a result, conservation programs often face a paradox in which increasingly sophisticated technologies are available, but the biological foundations required for their effective implementation are still lacking.

One strategy to overcome this limitation has been the use of model species as experimental platforms for developing reproductive technologies that can subsequently be adapted to related or threatened taxa. Under this perspective, reproductive physiology becomes more than a descriptive discipline; it becomes the starting point for building conservation technologies. By generating fundamental biological knowledge and progressively translating it into practical applications, long-term research programs can create integrated frameworks linking physiology, assisted reproduction, cryobiology, tissue preservation, and biobanking.

Located within the Caatinga biome, the Federal Rural University of the Semi-Arid (UFERSA) hosts one of the main research centers dedicated to wildlife reproductive biology in Brazil. Over the last twenty years, the Laboratory of Animal Germplasm Conservation (LCGA/UFERSA) and its collaborators have investigated the reproductive biology of multiple Neotropical species, particularly those associated with the Caatinga. What began as efforts to understand species-specific reproductive characteristics progressively evolved into a broader conservation-oriented research program encompassing reproductive physiology, assisted reproductive technologies, germplasm preservation, tissue culture, gonadal biobanking, and fertility restoration strategies.

Rather than presenting a simple chronological review of scientific outputs, this article synthesizes the conceptual evolution of this research program and discusses how model species have driven the development of conservation technologies. By examining two decades of studies spanning physiology, biotechnology, and biobanking, we propose a framework illustrating how reproductive research can be transformed into a practical conservation model for Neotropical wildlife and potentially for biodiversity conservation programs worldwide.

## Building the biological foundation

Every conservation technology begins with biology. During the first phase of the LCGA/UFERSA conservation program, efforts focused on generating baseline information regarding male and female reproductive physiology. Studies addressed reproductive anatomy, spermatogenesis, sperm maturation, ovarian follicular populations, estrous cycles, endocrine profiles, and reproductive microbiomes. Because it was impossible to investigate every wildlife species simultaneously, our strategy relied on identifying model species capable of representing broader taxonomic and physiological groups. This approach ultimately provided the biological foundation upon which assisted reproductive technologies, germplasm preservation protocols, and biobanking strategies were subsequently developed.

### Understanding male reproductive diversity

One of the major challenges in developing germplasm conservation strategies for wildlife species is the limited availability of fundamental reproductive information. Despite Brazil harboring one of the richest biodiversities worldwide, basic reproductive parameters remain poorly understood for many native species. Consequently, understanding species-specific reproductive physiology became a central component of our research program and the foundation upon which subsequent technological developments were built.

Among the species investigated, the collared peccary (*Pecari tajacu*) progressively emerged as the principal model for studies of male reproductive physiology in various other Neotropical species (Table 1). Long-term investigations involving numerous individuals established reference values for seminal characteristics (Peixoto et al., 2012), and enabled the validation of several analytical approaches, including sperm morphology (Sousa et al., 2013b), membrane functionality (Santos et al., 2013), sperm motility kinetics (Souza et al., 2016), sperm-binding ability to heterologous substrates (Campos et al., 2017), seminal plasma proteomics (Santos et al., 2014), organic and inorganic biochemical composition of this fluie (Moreira et al., 2019), and reproductive microbiota (Santos et al., 2020). Together, these studies generated one of the most comprehensive datasets currently available for a Neotropical wild mammal and established a benchmark for future investigations.

**Table 1:** Characteristics of semen and epididymal spermatozoa (collected by retrograde flushing and flotation) from the species studied by the Laboratory of Animal Germplasm Conservation (LCGA-UFERSA).

**Species**	**Collection method**	**Volume (μl)**	Concentration (sperm/mL)*	Number of sperm recovered*	**Motility (%)**	**Viability (%)**	**Functional membrane integrity (%)**	**Normal Morphology (%)**	**Reference**
Coati (*Nasua nasua*)	semen collection by electroejaculation	63	0.6 x 10^7^		68	72	79	85.3	Lima et al. (2009)
Collared peccaries (*Pecary tajacu*)		3.5 ± 3.8 x 10^3^	80 ± 70 x 10^7^		85.1 ± 10.7	86.3 ± 13.3	76.9 ± 13.2	79.9 ± 12.8	Peixoto et al. (2012)
Six-banded armadillo (*Euphractus sexcinctus*)		279.2 ± 48.6	75.7 ± 14.6 × 10^7^		65.8 ± 7.1	84.9 ± 4.2	59.5 ± 5.9	89.4 ± 1.5	Sousa et al. (2013a)
Agouti (*Dasiprocta leporina*)		252 ± 46	35.7 ± 61.2 x10^7^		80 ± 29.6	65.5 ± 26.1	68.5 ± 17.6	83.9 ± 8.6	Castelo et al. (2015b)
Agouti (*Dasiprocta leporina*)	epididymal sperm collection by retrograde flushing			1100 ± 300 × 10^6^	91.5 ± 5.0	59.1 ± 3.8	71.4 ± 4.8	85.1 ± 1.3	Silva et al. (2011)
Collared peccaries (*Pecary tajacu*)				635.9 ± 188.6 × 10^6^	57.8 ± 13.2	68.2 ± 5.7	60.3 ± 7.3	52.3 ± 3.1	Bezerra et al. (2014)
Spix’s yellow-toothed cavies (*Galea spixii*)				345.1 ± 86.3 × 10^6^	56.1 ± 7.1	56.1 ± 6.1	61.5 ± 6.9	58.8 ± 4.4	Silva et al. (2017)
Collared peccaries (*Pecary tajacu*)	epididymal sperm collection by flotation			1096.1 ± 462.5 × 10^6^	64.4 ± 10.6	63.1 ± 5.2	64.0 ± 5.2	57.3 ± 4.4	Bezerra et al. (2014)
Spix’s yellow-toothed cavies (*Galea spixii*)				514.8 ± 202.3 × 10^6^	72.2 ± 9.1	64.2 ± 4.7	51.8 ± 6.3	55.3 ± 5.2	Silva et al. (2017)
Agouti (*Dasiprocta leporina*)				1375 ± 244.1 × 10^6^	76.8 ± 6.2	83.5 ± 3.4	83.3 ± 3.9	87.3 ± 1.9	Dantas et al. (2022a, b)
*R*ed-nosed-mouse *(Wiedomys pyrrhorhinos*)				31.4 ± 9.9 x10^6^	87.6 ± 2.5	56.0 ± 11.9	80.3 ± 2.8	94.2 ± 0.9	Matos (2026)

Complementary studies in red-rumped agoutis (*Dasyprocta leporina* – Arroyo et al., 2017; Dantas et al., 2022a, b), Spix’s yellow-toothed cavies (*Galea spixii* - Santos et al., 2012), and red-nosed rats (*Wiedomys pyrrhorhinos* – Matos, 2026) expanded our understanding of spermatogenesis and sperm maturation. These investigations revealed that reproductive strategies among Neotropical mammals are far more diverse than previously assumed and frequently differ from patterns described in domestic species.

Our comparative approach also extended to other taxa and other biomes, like Pantanal and Cerrado. Investigations involving lowland tapirs (*Tapirus terrestris*) characterized reproductive behavior, testosterone profiles, and testicular development across different life stages and environmental conditions (Brasil et al., 2022). In parallel, studies on reproductive physiology in other groups like the birdes, focused on reproductive physiology of the greater rhea (*Rhea americana –*Bezerra et al., 2023a, b), and reinforced the importance of comparative reproductive biology.

Given the predicted impacts of climate change on wildlife populations, increasing attention was also directed toward understanding how environmental variables influence reproductive performance. Studies in peccaries (Maia et al., 2019), agoutis (Dantas et al., 2024), and even in honeybees (Morais et al., 2024) demonstrated that rainfall, humidity, temperature, and solar radiation can significantly affect reproductive parameters, highlighting the potential vulnerability of reproductive processes to future environmental changes.

Collectively, these investigations established baseline reproductive parameters for multiple wildlife species and revealed remarkable reproductive diversity among Neotropical animals. More importantly, they demonstrated that assumptions derived from domestic species cannot always be directly extrapolated to wildlife taxa. However, understanding reproductive physiology alone was insufficient for conservation purposes. The next challenge was to transform biological knowledge into practical reproductive tools.

### Unraveling female reproductive complexity for conservation

Understanding female reproductive biology represents a fundamental step toward the development of effective conservation strategies for wildlife species. Knowledge regarding ovarian dynamics, reproductive endocrinology, follicular reserve, and reproductive microbiota is essential not only for advancing assisted reproductive technologies but also for establishing fertility preservation and biobanking programs.

A significant component of our research efforts has focused on characterizing the ovarian biology of native species from the Caatinga biome and identifying physiological parameters that could support future conservation initiatives (Campos et al., 2016; Lima et al., 2025). Substantial variation in ovarian reserve size and follicular organization was observed among species, revealing remarkable diversity in ovarian biology across Neotropical mammals. The total follicular population ranged from approximately 5,000 PAFs per ovarian pair in the red-rumped agouti (Santos et al., 2018) to 15,567 PAFs in the six-banded armadillo (*Euphractus sexcinctus*) (Lima et al., 2025) and 33,743 follicles in the collared peccary (Lima et al., 2013). In most species, primordial follicles represented the predominant follicular category, reflecting a large dormant ovarian reserve. An interesting exception was observed in the Spix’s yellow-toothed cavy (*Galea spixii*), where the ovarian reserve was characterized by a predominance of primary follicles and a considerably smaller total follicular population of approximately 200 PAFs per ovarian pair (Praxedes et al., 2017). These findings provided essential baseline information for the development of ovarian tissue preservation protocols and future female germplasm banking strategies.

Beyond static ovarian morphology, our investigations sought to understand the dynamic regulation of female reproductive function. Through the integration of behavioral observations, clinical examinations, vaginal cytology, ovarian ultrasonography, and endocrine monitoring, we characterized estrous cycle dynamics in several species. These studies demonstrated that cycle length varies considerably among taxa, ranging from 21.5 days in collared peccaries (Maia et al., 2014a) and 23.5 days in six-banded armadillos (Campos et al., 2016) to 28.2 days in red-rumped agoutis (Campos et al., 2015). Such information proved indispensable for the subsequent development of hormonal monitoring protocols, reproductive management strategies, and assisted reproduction techniques.

More recently, our research has expanded toward the investigation of reproductive microbiomes as an additional component of female reproductive physiology. Initial studies in collared peccaries demonstrated substantial qualitative variation in vaginal bacterial communities among females at different reproductive stages, despite relatively stable proportions of major bacterial groups (Cavalcante et al., 2025). These findings suggest that reproductive microbiota may represent an additional source of physiological variation influencing reproductive health and fertility, opening new perspectives for future conservation-oriented reproductive studies.

Together, these investigations established one of the most comprehensive datasets currently available on female reproductive biology in Neotropical wildlife. More importantly, they demonstrated that ovarian morphology, follicular reserve, endocrine regulation, and reproductive microbiota are closely interconnected components of female reproductive function. The knowledge generated during this phase provided the biological foundation necessary for subsequent advances in ovarian tissue preservation, xenotransplantation, in vitro follicle culture, and female germplasm banking. Thus, beyond their immediate value for species conservation, these studies positioned several Neotropical mammals as strategic biological models for the development of emerging reproductive technologies applicable to wildlife conservation worldwide.

### Building species specific reproductive tools

Once the fundamental aspects of reproductive physiology had been characterized, the next challenge was to transform biological knowledge into practical conservation tools. The development of reproductive technologies for wildlife conservation required not only an understanding of reproductive biology but also the establishment of species-specific methodologies for sample collection, evaluation, and management.

Initial studies focused on optimizing anesthetic and semen collection protocols for multiple wildlife species as the collared peccary (Souza et al., 2009), the ring tail coati (Barros et al., 2009) and the six-banded armadillo (Sousa et al., 2016a). Comparative investigations demonstrated that methodologies successfully applied in one species could not always be directly transferred to another, reinforcing the importance of species-specific solutions. The red-rumped agouti represents a notable example of this limitation. Despite the evaluation of different anesthetic regimens, electroejaculation devices, and stimulation protocols, more than 60% of collected samples remained azoospermic (Castelo et al., 2015b. This concept became one of the central principles guiding subsequent advances in wildlife reproductive biotechnology.

Alternative collection strategies, including urethral catheterization as demonstrated for the crab-eating fox (*Cerdocyon thous*) through a collaboration with Reprocon institute (Silva et al., 2022a), as well as the epididymal sperm recovery applied to various species native from the Caatinga (Bezerra et al., 2014; Silva et al., 2017), expanded opportunities for germplasm rescue, particularly in species for which conventional semen collection remained difficult or inefficient. Importantly, these developments were not restricted to mammals, as similar efforts contributed to the understanding and preservation of reproductive material from birds (Bezerra et al., 2023a, b) and other taxa as the bees (Dantas et al., 2026).

Throughout this process, the collared peccary progressively became the primary experimental platform for developing and validating reproductive technologies that were subsequently adapted to other wildlife species. In this sense, the species served not only as a conservation target but also as a biological model driving technological innovation.

## Model species driving conservation technologies

A defining feature of the program was the adoption of model species. The collared peccary became the primary model for reproductive technologies, while agoutis contributed to ovarian biology and epididymal maturation studies. Spix’s yellow-toothed cavy served as a model for folliculogenesis and spermatogenesis, jaguars for validation of cryopreserved germplasm through embryo production, and tapirs for reproductive endocrinology. On the other hand, the xenartras them represent the new frontier, since because they do not have phylogenetic proximity to any other species, they need the development of their own study methods, and the development of unprecedent strategies for the conservation of their germplasm. These strategies accelerated technology development and facilitated translation to threatened taxa.

### From sperm cryopreservation to testicular biobanking

Male reproductive fitness plays a fundamental role in population viability and genetic diversity maintenance (Rankin and Kokko, 2007). Consequently, the development of strategies for preserving male germplasm has been a central component of the conservation framework established by LCGA/UFERSA, encompassing both mature gametes and testicular tissues (Santos et al., 2025a). Over the last two decades, our efforts have progressively evolved from conventional sperm cryopreservation toward integrated testicular biobanking approaches aimed at safeguarding present and future reproductive potential.

Among currently available conservation technologies, sperm cryopreservation remains the most mature and widely applicable strategy for long-term preservation of male genetic resources in wildlife species (Prieto et al., 2014). The methodology is relatively well established and involves the collection, evaluation, processing, cryostorage, and subsequent thawing of sperm samples (Silva et al., 2021; Moreira et al., 2023). However, while sperm banking remains the cornerstone of male germplasm preservation, emerging technologies based on reproductive tissues are increasingly expanding the scope of wildlife biobanking (Silva et al., 2020a).

The collared peccary has served as the primary experimental model for the development and refinement of sperm cryopreservation protocols in our laboratory. Since the first successful freezing protocol described by Castelo et al. (2010), numerous methodological improvements have been achieved (Figure 1), resulting in substantial gains in post-thaw sperm quality. Among the main strategies developed, notable approaches include the use of new extenders, such as powdered coconut water (Silva et al., 2012a) and Aloe vera (Souza et al., 2016), as well as the evaluation of different cryopreservation curves, straw volumes, and thawing rates (Silva et al., 2013), low-density lipoproteins (Souza et al., 2015), additives such as Equex STM (Bezerra et al., 2019), antimicrobials (Moreira et al., 2022), and sodium dodecyl sulfate (Moreira et al., 2023), in addition to refrigerated transport at 5 °C (Santos et al., 2024). Additionally, protocols for the recovery and cryopreservation of epididymal spermatozoa were also established (Bezerra et al., 2018). More recently, individual variation in cryotolerance was identified, allowing males to be classified as either good or poor semen freezers (Moreira et al., 2025). These advances have culminated in protocols capable of maintaining approximately 50% sperm motility after thawing, even following short-term refrigerated transport prior to cryopreservation (Santos et al., 2024).

**Figure 1:**
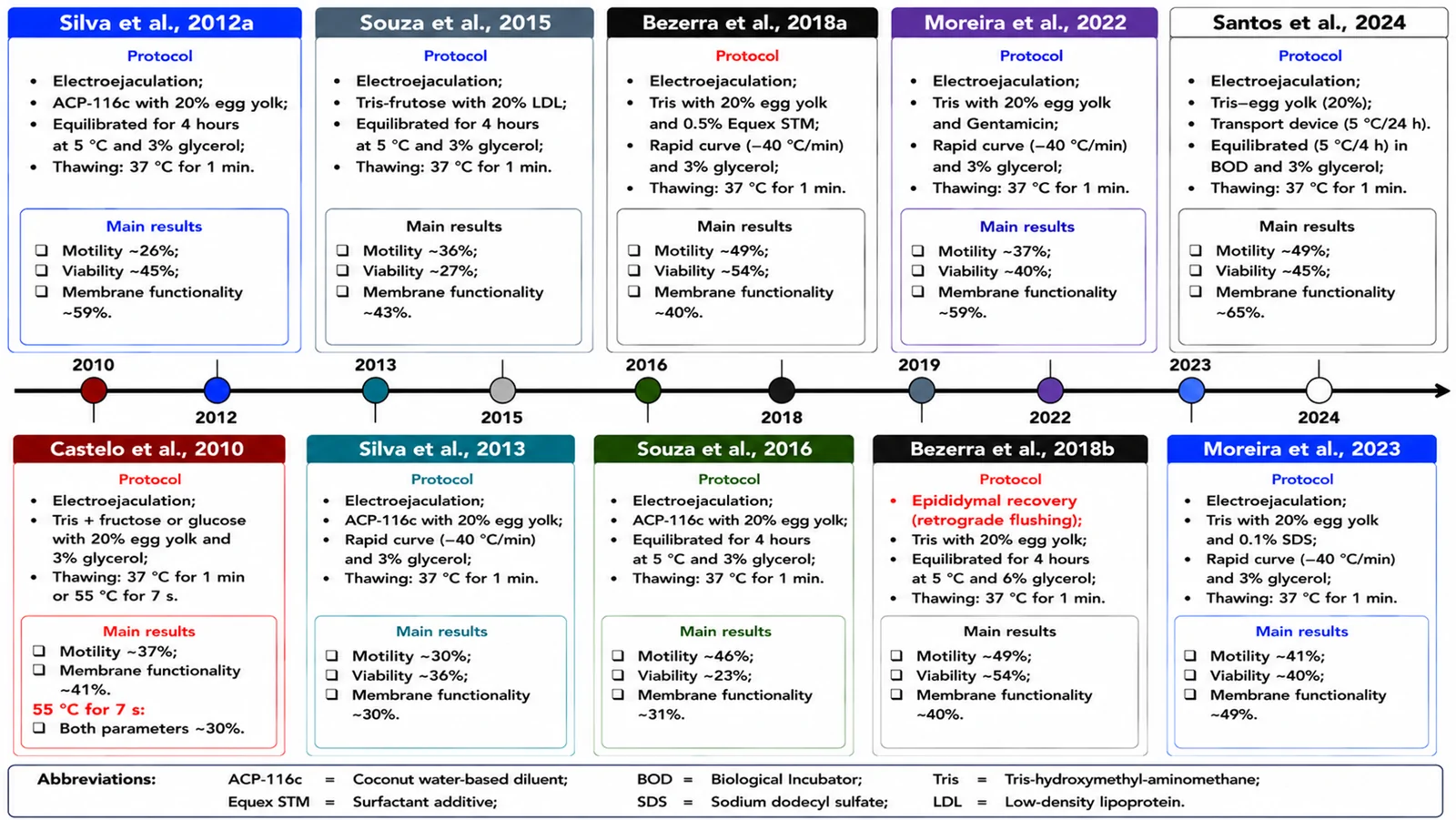
Timeline illustrating the evolution of sperm cryopreservation protocols for collared peccaries (*Pecari tajacu*) developed between 2010 and 2024.

The knowledge generated using collared peccaries as a model species accelerated the development of cryopreservation strategies for other wildlife taxa (Figure 2). In wild rodents, for example, sperm cryopreservation protocols have been established for the red-rumped agouti (*Dasyprocta leporina*), using spermatozoa obtained either by electroejaculation (Castelo et al., 2023) or recovered from the epididymis (Silva et al., 2012b; Castelo et al., 2015a). Relevant results have also been obtained in the yellow-toothed cavy (*Galea spixii*), particularly using epididymal spermatozoa (Silva et al., 2018; Moreira et al., 2021; Brito et al., 2026). Nevertheless, these studies also highlighted the importance of species-specific reproductive characteristics. In six-banded armadillos (*Euphractus sexcinctus*), for example, spermatozoa naturally aggregate through concave and convex membrane regions, forming rouleaux-like structures embedded in a viscous matrix. This unique biological feature has posed significant challenges for the establishment of efficient cryopreservation protocols (Sousa et al., 2016b), illustrating how reproductive diversity frequently demands tailored technological solutions.

**Figure 2:**
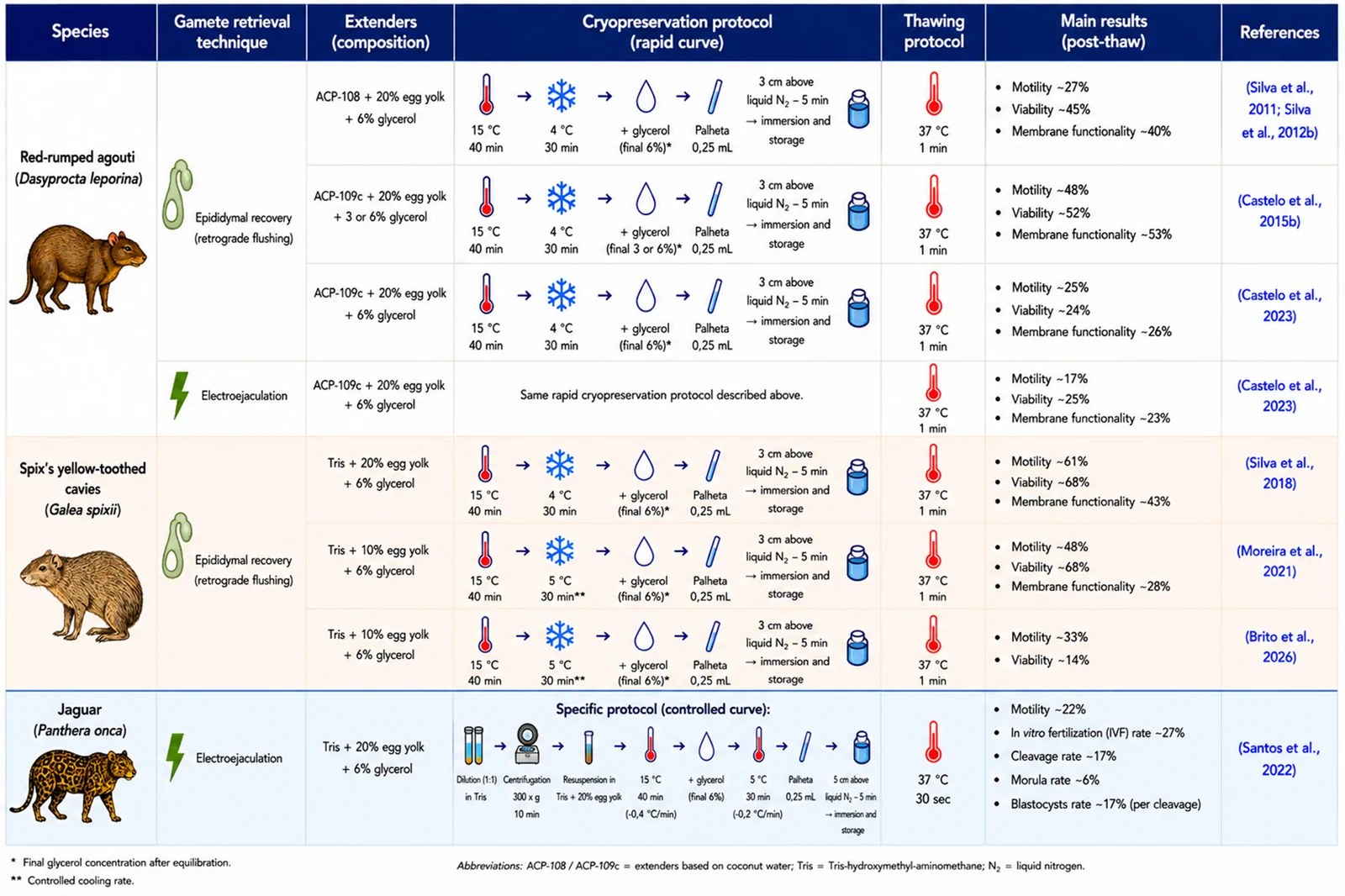
Advances in sperm cryopreservation protocols developed by the LCGA for wild mammal species.

In contrast, significant progress has been achieved in species of high conservation concern through effective partnerships with Laboratory of Carnivore Conservation of State University of Ceará and Laboratory on Animal Biotechnology – LBA/UFERSA. In jaguars (*Panthera onca*), semen collected by electroejaculation was successfully cryopreserved while maintaining approximately 50% post-thaw sperm motility (Silva et al., 2020b). More importantly, the functional competence of cryopreserved sperm was validated through heterologous *in vitro* embryo production using domestic cat oocytes (Santos et al., 2022). This achievement represented a critical milestone, demonstrating that biobanked germplasm can support downstream reproductive outcomes and confirming the practical value of cryopreservation for conservation purposes.

Our research program has also expanded beyond mammals. In greater rheas (*Rhea americana*), initial cryopreservation studies generated valuable information regarding sperm preservation in ratites, although post-thaw motility remains relatively low and further protocol optimization is required (Bezerra et al., 2026). Similarly, studies in Africanized honeybees (*Apis mellifera*) demonstrated the feasibility of sperm cryopreservation using Tris-based extenders supplemented with DMSO, generating information that may contribute both to germplasm banking and artificial insemination programs in this economically and ecologically important species (Morais et al., 2023).

Although cryopreservation remains the preferred strategy for long-term germplasm preservation, its application under field conditions is often limited by logistical constraints (Campbell et al., 2021). Therefore, complementary approaches have been investigated, including short-term refrigeration protocols for sperm and reproductive tissues (Santos et al., 2025b, 2026). These strategies are particularly relevant when sample collection occurs in remote locations, where immediate cryopreservation may not be feasible (Krishnakumar et al., 2013).

A major conceptual advance in recent years has been the transition from preserving isolated spermatozoa to preserving complete testicular tissues, in a long term collaboration with Smithsonian Conservation Biology Institute. Unlike sperm banking, testicular biobanking preserves not only mature gametes but also spermatogonial stem cells and supporting somatic cells, thereby maintaining the potential for future sperm production (Silva et al., 2020a). Such tissues can subsequently be subjected to in vitro culture, xenotransplantation, or emerging reproductive technologies aimed at restoring spermatogenesis and generating functional gametes (Yokonishi et al., 2013; Higaki et al., 2018).

Using the collared peccary as a model species, our group established some of the first protocols for cryopreservation of both adult and prepubertal testicular tissues in Neotropical wildlife (Silva et al., 2019; Silva et al., 2021, 2025a). These studies demonstrated that different freezing and vitrification approaches can maintain substantial levels of cellular viability, typically ranging from 40% to 70% depending on the protocol and developmental stage. Building upon these advances, subsequent investigations demonstrated that supplementation with glial cell line-derived neurotrophic factor improved cell survival and proliferation during in vitro culture of prepubertal testicular tissue, maintaining approximately 65% viable cells after 28 days (Silva et al., 2024). More recently, long-term organotypic culture systems capable of supporting testicular fragments for approximately 54 days—the physiological duration of spermatogenesis in collared peccaries—have been established, representing an important step toward future *in vitro* spermatogenesis (Pereira et al., 2025).

Additional advances included the development of protocols for refrigerated transport of testes prior to processing, enabling the recovery of highly viable tissues after storage at 5 °C for up to 24 hours (Loia et al., 2025). Furthermore, testicular fragments refrigerated before vitrification maintained acceptable viability and apoptosis levels following warming (Santos et al., 2025b; Santos et al., 2026), expanding opportunities for field-based germplasm rescue and biobanking.

The applicability of testicular tissue preservation has also been demonstrated in other species. In red-rumped agoutis (*Dasyprocta leporina*), the first successful attempts at testicular tissue cryopreservation yielded approximately 45% viable cells after warming (Silva et al., 2022b), reinforcing the feasibility of extending these approaches to additional wildlife taxa.

Collectively, these studies illustrate the progressive evolution of our conservation strategy—from sperm preservation to testicular biobanking. More importantly, they demonstrate how advances in reproductive physiology, cryobiology, tissue culture, and germplasm preservation can be integrated into a comprehensive framework aimed at preserving not only current fertility, but also future reproductive opportunities for wildlife populations.

### Harnessing female reproductive physiology to enable assisted reproduction

The successful application of assisted reproductive technologies depends fundamentally on the ability to control and predict ovarian function. Therefore, one of the first objectives of our research program was to establish reliable protocols for estrous synchronization and ovarian stimulation in Neotropical wildlife species, particularly the collared peccary, which became the principal model for female reproductive studies.

Initial synchronization protocols based on prostaglandin administration successfully induced estrus in most females, although considerable variability was observed in the timing of estrous expression (Maia et al., 2014b). Subsequent protocols using gonadotropin combinations (eCG/hCG) produced more predictable ovarian responses and enabled the first attempts at artificial insemination in the species (Peixoto et al., 2019). Although these initial insemination trials did not result in offspring, they generated valuable information regarding endocrine regulation, ovarian responsiveness, and luteal function, highlighting the importance of species-specific optimization of hormonal protocols.

Importantly, these studies provided the physiological foundation required for subsequent advances in *in vitro* embryo production. Through collaboration with LBA/UFERSA, hormonal stimulation protocols enabled the recovery of developmentally competent oocytes and supported the establishment of systems for oocyte maturation (Borges et al., 2018), parthenogenetic embryo production (Borges et al., 2020), and heterologous (Santos et al., 2023) *in vitro* fertilization. These achievements demonstrated how knowledge of ovarian physiology can be directly translated into reproductive technologies with potential applications for conservation and genetic management.

### Ovarian tissue biobanking: expanding conservation opportunities

While assisted reproductive technologies provide opportunities for immediate reproductive management, long-term biodiversity conservation requires strategies capable of preserving female genetic resources beyond the reproductive lifespan of individual animals. In this context, ovarian tissue biobanking emerged as one of the most promising components of our conservation program (Table 2).

**Table 2:** Timeline of major publications on female germplasm conservation developed by the Laboratory of Animal Germplasm Conservation (LCGA-UFERSA) between 2010 and 2025.

Species	Objective	Methodology	Main Quantitative Results	Scientific Contribution	Authors
*Pecari tajacu*	Characterization of ovarian preantral follicle population	Histology and morphometry	>33,000 follicles/ovary; 92% primordial follicles; >94% normal follicles	First detailed description of ovarian reserve in collared peccaries	Lima et al. (2013)
*Galea spixii*	Characterization and vitrification of ovarian follicles	Histology, SSV, TEM	94.6% normal follicles before vitrification; ~70% preserved after warming	First ovarian tissue vitrification protocol for the species	Praxedes et al. (2017)
*Pecari tajacu*	Evaluating ovarian tissue vitrification comparing different cryoprotectants	Histology and viability assays	High follicular survival following vitrification. EG showed highest follicular preservation rates	Demonstrated feasibility of ovarian tissue cryobanking	Lima et al. (2019)
*Pecari tajacu*	Validating Ovarian Tissue Cryosystem (OTC)	Morphology, viability, cell proliferation capacity and apoptosis	Morphology, viability and proliferation comparable to fresh tissue; Low apoptotic rates and high membrane integrity	First biosafe closed system validated for wildlife ovarian tissue	Campos et al. (2019)
*Dasyprocta leporina*	Evaluating follicle survival after xenografting of vitrified ovarian tissue	Morphology, cell proliferation and the occurrence of DNA fragmentation	Xenografted agouti ovarian tissue, fresh or vitrified, is able to promote the return of ovarian activity in ovariectomised SCID C57B1/6 mice		Praxedes et al. (2018)
*Dasyprocta leporina*	Characterization of ovarian morphology	Histology and morphometry	Description of follicular categories and ovarian architecture	Reproductive baseline data for agoutis	Santos et al. (2018)
*Dasyprocta leporina*	Assessment of advanced ovarian tissue preservation approaches	Cryobiology and follicular analyses	Improved post-thaw follicular quality	Refinement of cryobanking strategies	Praxedes et al. (2023)
Multiple species	Reviewing gonadal tissue preservation strategies	Conservation biotechnology review	Consolidation of protocols developed in wildlife species	Established framework for wildlife gonadal biobanking	Silva et al. (2022c)
Multiple species	Conservation-oriented reproductive technologies	Comprehensive review	Expansion of fertility preservation strategies	Consolidated LCGA as an international reference in wildlife germplasm conservation	Silva et al. (2025b)

Unlike isolated oocytes, ovarian cortical fragments contain hundreds or even thousands of preantral follicles, each representing a potential future offspring. Consequently, ovarian tissue preservation greatly expands opportunities for genetic rescue, particularly in juvenile, senescent, pregnant, or unexpectedly deceased females. Moreover, ovarian biobanking creates opportunities for the future application of emerging technologies, including in vitro folliculogenesis, ovarian tissue transplantation, and stem-cell-based reproductive approaches (Campos et al., 2019a).

Using the collared peccary as a model species, our group established one of the first systematic research programs on ovarian tissue preservation in Neotropical wildlife. Initial studies focused on short-term ovarian transport and storage, followed by the development of vitrification protocols capable of maintaining high follicular survival and viability (Lima et al., 2014; Lima et al., 2019). Subsequent studies demonstrated that closed vitrification systems such as the Ovarian Tissue Cryosystem (OTC), particularly when combined with ethylene glycol, could preserve follicular morphology, viability, proliferative activity, and apoptotic status at levels comparable to fresh controls (Campos et al., 2019b).

The knowledge generated in collared peccaries subsequently served as the basis for adapting ovarian tissue preservation protocols to other wildlife species, including Spix’s yellow-toothed cavies (Praxedes et al., 2017) and red-rumped agoutis (Praxedes et al., 2020). Among these advances, perhaps the most remarkable achievement was the demonstration that vitrified ovarian tissue from agoutis could be successfully xenografted into immunosuppressed mice, restoring endocrine activity and supporting follicular development, ovulation, and corpus luteum formation after transplantation (Praxedes et al., 2018). This study provided compelling evidence that ovarian tissue biobanking can preserve not only tissue viability but also long-term reproductive competence.

More recently, complementary studies investigating follicle culture systems have further expanded the potential applications of ovarian biobanking. The supplementation of culture media with FSH, GDF-9, and BMP-15 has improved follicular survival and development in preserved ovarian tissues (Lima et al., 2018; Gomes et al., 2020; Campos et al., 2021), creating new opportunities for future applications involving in vitro folliculogenesis and fertility restoration.

Added up, these studies illustrate the evolution of female germplasm conservation from endocrine control and assisted reproduction toward comprehensive ovarian tissue biobanking. More importantly, they demonstrate how physiological knowledge, reproductive biotechnology, and tissue preservation can be integrated into a unified framework for the long-term conservation of genetic diversity in wildlife populations.

## Lessons learned after twenty years

The trajectory described throughout this review extends far beyond the development of individual reproductive technologies. It represents two decades of continuous efforts to understand the reproductive biology of Neotropical wildlife and to translate this knowledge into practical conservation tools. Throughout this process, several scientific, technical, and strategic lessons emerged (Figure 3), shaping not only our research program but also our perspective on the role of reproductive science in biodiversity conservation.

**Figure 3:**
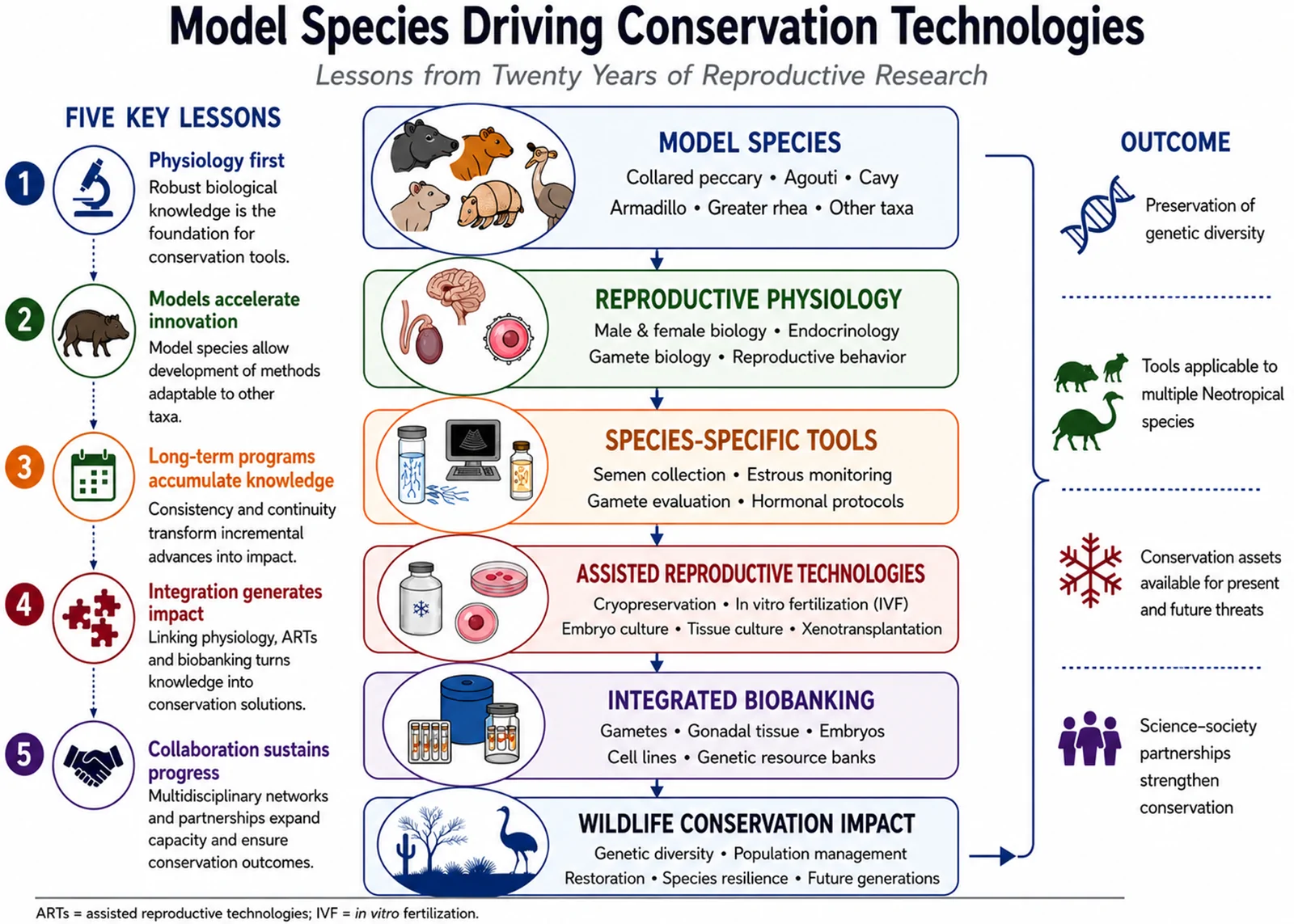
Framework summarizing the conceptual evolution of the reproductive research program developed by the Laboratory of Animal Germplasm Conservation (LCGA/UFERSA) over the last two decades, illustrating how model species served as platforms for the development of conservation technologies applicable to Neotropical wildlife.

The first and perhaps most important lesson is that reproductive physiology remains the foundation of all conservation technologies. Every significant advance achieved by our group originated from a detailed understanding of species-specific reproductive characteristics. Studies on spermatogenesis, sperm maturation, seminal physiology, follicular dynamics, ovarian reserve, reproductive endocrinology, and reproductive microbiota provided the biological framework necessary for the development of cryopreservation protocols, assisted reproductive technologies, and biobanking strategies. Repeatedly, we observed that technologies developed without a thorough understanding of reproductive physiology were less effective or required substantial adaptation before becoming applicable to wildlife species.

A second lesson concerns the strategic importance of model species. The use of species such as the collared peccary, red-rumped agouti, Spix’s yellow-toothed cavy, six-banded armadillo, and greater rhea allowed the gradual development and validation of technologies that would be difficult or ethically challenging to test directly in threatened species. These models provided experimental platforms for advancing knowledge on reproductive physiology, cryobiology, tissue preservation, and *in vitro* culture systems, while simultaneously generating valuable information for the conservation of the species themselves. This approach demonstrated that model species can serve as powerful drivers of technological innovation in wildlife conservation.

The third lesson is that long-term research programs generate cumulative gains that isolated projects cannot achieve. Many of the advances described in this review were only possible because they resulted from sequential studies conducted over many years. Initial investigations focused on basic reproductive characterization, which subsequently supported the development of collection methods, cryopreservation protocols, hormonal manipulation strategies, tissue preservation systems, and ultimately integrated biobanking approaches. The progression from sperm collection to testicular biobanking, and from ovarian characterization to ovarian tissue preservation and xenotransplantation, exemplifies how scientific advances often depend on the accumulation of knowledge across multiple generations of projects, students, and collaborations.

A fourth lesson emerged from the realization that conservation success depends less on maximizing the performance of individual technologies and more on integrating complementary approaches into a coherent conservation framework. Sperm cryopreservation alone cannot guarantee genetic preservation, just as ovarian tissue banking, hormonal manipulation, or embryo production are insufficient when applied in isolation. The most significant advances occurred when reproductive physiology, cryobiology, tissue culture, endocrinology, reproductive microbiology, assisted reproduction, and biobanking were combined into an integrated strategy capable of addressing different conservation challenges. This integrated perspective ultimately led to the concept of a comprehensive germplasm conservation framework for wildlife species.

## Conclusions and perspectives

Finally, our experience reinforced the inherently multidisciplinary nature of conservation science. The development of effective conservation strategies required the collaboration of reproductive physiologists, veterinarians, geneticists, cryobiologists, microbiologists, ecologists, wildlife managers, and conservation practitioners. Equally important was the contribution of undergraduate students, graduate students, postdoctoral fellows, and national and international collaborators who collectively helped build the scientific network supporting these advances. In this sense, one of the most important outcomes of the program may not be a specific technology or protocol, but rather the human capacity and collaborative infrastructure developed throughout the process.

Taken together, these lessons illustrate how reproductive biotechnology can evolve from a collection of isolated techniques into a strategic conservation tool. The studies summarized in this review generated extensive information on the reproductive physiology of wildlife species from the Caatinga biome, including seminal characteristics, sperm function, seminal plasma composition, reproductive microbiota, ovarian reserve, follicular dynamics, and endocrine regulation. Beyond expanding biological knowledge, these investigations established the scientific basis for germplasm preservation, assisted reproduction, and biobanking initiatives aimed at maintaining genetic diversity in wildlife populations.

Looking forward, future efforts should focus on increasing the efficiency, reproducibility, and scalability of existing technologies, expanding their application to a broader range of threatened species, and strengthening the integration between *ex situ* and *in situ* conservation programs. The Caatinga biome and its model species have demonstrated how long-term investment in reproductive science can generate practical conservation solutions. However, despite the considerable progress achieved over the past twenty years, the greatest opportunities may still lie ahead. Emerging technologies such as organoids, *in vitro* gametogenesis, stem-cell-based approaches, conservation genomics, and artificial intelligence are likely to reshape the future of wildlife conservation. In many ways, the journey described here represents not a conclusion, but the beginning of a new phase in the application of reproductive technologies for biodiversity conservation.

## Data Availability

Data that support the findings of this study are available from the authors upon request.
